# Effect of Fibroblast Growth Factor 2 on Equine Synovial Fluid Chondroprogenitor Expansion and Chondrogenesis

**DOI:** 10.1155/2016/9364974

**Published:** 2015-12-29

**Authors:** Marta Bianchessi, Yuwen Chen, Sushmitha Durgam, Holly Pondenis, Matthew Stewart

**Affiliations:** ^1^Department of Veterinary Clinical Medicine, University of Illinois, Urbana, IL 61802, USA; ^2^QPS Taiwan, Center of Toxicology and Preclinical Sciences, No. 103, Lane 169, Kangning Street, Xizhi District, New Taipei City 221, Taiwan

## Abstract

Mesenchymal stem cells have been identified in the synovial fluid of several species. This study was conducted to characterize chondroprogenitor (CP) cells in equine synovial fluid (SF) and to determine the effect of fibroblast growth factor 2 (FGF-2) on SF-CP monolayer proliferation and subsequent chondrogenesis. We hypothesized that FGF-2 would stimulate SF-CP proliferation and postexpansion chondrogenesis. SF aspirates were collected from adult equine joints. Colony-forming unit (CFU) assays were performed during primary cultures. At first passage, SF-cells were seeded at low density, with or without FGF-2. Following monolayer expansion and serial immunophenotyping, cells were transferred to chondrogenic pellet cultures. Pellets were analyzed for chondrogenic mRNA expression and cartilage matrix secretion. There was a mean of 59.2 CFU/mL of SF. FGF-2 increased the number of population doublings during two monolayer passages and halved the population doubling times. FGF-2 did not alter the immunophenotype of SF-CPs during monolayer expansion, nor did FGF-2 compromise chondrogenesis. Hypertrophic phenotypic markers were not expressed in control or FGF-2 groups. FGF-2 did prevent the development of a “fibroblastic” cell layer around pellet periphery. FGF-2 significantly accelerates in vitro SF-CP expansion, the major hurdle to clinical application of this cell population, without detrimentally affecting subsequent chondrogenic capacity.

## 1. Introduction

Articular cartilage is a highly specialized connective tissue, responsible for equilibrating loads across joint surfaces and minimizing friction during joint motion. Cartilage is an alymphatic, avascular, and aneural tissue, with a comparatively low cellular density. These characteristics limit the intrinsic reparative capacity of articular cartilage [1]. Current surgical treatments for articular cartilage injuries [2–4] do not reliably restore a functional and phenotypically stable cartilage matrix. Further, in vitro expansion of chondrocytes, prior to reimplantation into cartilage lesions, compromises the specialized phenotype of these cells [5, 6].

Mesenchymal stem cells (MSCs) represent a promising alternative resource for cartilage repair, given their chondrogenic potential, capacity for considerable proliferative expansion, ease of access, and immunogenic properties. The majority of initial research on stem cell chondrogenesis has been carried out using bone marrow-derived stem cells [7, 8], but it is now well recognized that progenitor cells exist in most tissues and body fluids, albeit in very low numbers, and that the chondrogenic capacities of these progenitor cell populations vary considerably [9–14]. The majority of MSC populations undergo chondrogenesis that culminates in a hypertrophic phenotype [8, 10, 15–17], not optimal for articular cartilage repair.

Several recent studies, utilizing synovial fluid aspirates from a range of species, have demonstrated that progenitor cells can be isolated from synovial fluid (SF-CP), expanded in vitro [18–22] and, under appropriate culture conditions, induced to express a nonhypertrophic chondrogenic phenotype that is more consistent with articular chondrocyte characteristics [19, 23–26]. Consistently, SF-CP concentrations are increased in arthritic conditions [18–22], suggesting a role for these cells in host responses to joint trauma and/or degeneration. Accepting their phenotypic suitability, the very low numbers of these cells in synovial fluid [19, 22, 23, 26] and intrinsic limits to proliferation [20, 27] represent major obstacles to potential clinical applications of SF-CPs [20, 28, 29].

Fibroblast growth factor 2 (FGF-2), also known as basic fibroblast growth factor, is a potent mitogen in many cell types and also increases chondrogenesis and cartilage matrix formation in some progenitor populations [30–32]. The purpose of this study was to determine the effect of FGF-2 on equine SF-CP monolayer expansion and subsequent chondrogenic differentiation. We hypothesized that FGF-2 will stimulate SF-CP proliferation and improve postexpansion chondrogenesis.

## 2. Materials and Methods

### 2.1. Collections

This study was conducted with the approval of the University of Illinois' IACUC. Synovial fluid samples were collected aseptically from the tibiotarsal or metacarpotarsophalangeal joints of young adult horses (18 Standardbreds, two Thoroughbreds, and seven Quarter horses). There were 15 fillies/mares, four colts/stallions, and 8 geldings, with an age range of 2–4 years. The synovial aspirates were collected immediately prior to arthroscopy for removal of osteochondral lesions. The joints had minimal clinical or arthroscopic evidence of osteoarthritis.

### 2.2. Cell Culture

Two mL of synovial fluid was plated in 10 mL of low-glucose Dulbecco modified Eagle medium (DMEM) supplemented with 10% fetal bovine serum, 100 U of sodium penicillin/mL, and 100 *μ*g of streptomycin sulfate/mL. The primary cultures were incubated at 37°C in 5% CO_2_ with 90% humidity. Colony-forming units (CFU), defined as focal clusters of 25 or more cells (reflecting four or more cell divisions), were monitored in each dish during the first seven days in culture and were counted on day 7.

### 2.3. Cell Expansion

The primary monolayers were trypsinized at approximately 80% confluence, counted, and replated at 1 × 10^4^ cells/cm^2^. Cell viability was determined by trypan blue exclusion. First passage cells were maintained in growth medium (as above) or in medium supplemented with 100 ng of FGF-2/mL. In a previous study, this FGF-2 dose was found to optimally stimulate chondrogenesis of equine bone marrow-derived MSCs [32]. The medium was changed every 2 to 3 days, until 80% confluence. Replating was continued for two passages, to generate sufficient cell numbers for subsequent chondrogenesis experiments. The population doublings during each passage were calculated using the following formula: Log_2_ (harvested cell number/seeded cell number). The population doubling times during each passage were calculated dividing the time of each passage by the population doubling value.

### 2.4. Immunophenotypic Analysis

Flow cytometry was used to evaluate the SF-CP immunophenotype (CD29, CD44, and CD90) during monolayer expansion, following previously published recommendations [33, 34]. CD45 was included as a negative control for hematopoietic progenitors. At each passage, 2 × 10^6^ SF-CPs were resuspended in DMEM media with 1% BSA [34]. The following antibodies were used according to the manufacturers' recommendations: anti-human conjugated anti-CD29-Alexa 488 (BioLegend, San Diego, CA); anti-horse conjugated anti-CD44-RPE (AbD Serotec, BioRad, Hercules, CA); anti-horse nonconjugated anti-CD90-Alexa 647 (Accurate Chemical and Scientific Corporation, Westbury, NY); and anti-human conjugated anti-CD45-Alexa 488 (AbD Serotec, BioRad, Hercules, CA) [34]. Bone marrow-derived MSCs and chondrocytes were used as biological controls. The following filters were used in a flow cytometry analyzer (Accuri C6, BD Biosciences, CA) to isolate the emission wavelength of the conjugated fluorochromes: FL-1 (510 nm and 545 nm wavelengths of light) for CD29 (519 nm emission) and CD45 (519 nm emission), FL-2 (560–580 nm wavelength) for CD44 (578 nm emission), and FL-4 (665–695 nm wavelength) for CD90 (668 nm emission). After the emission analysis on “FCS Express (Flow Research Edition),” data were expressed as “percentage of deviation from the control antibody groups.”

### 2.5. In Vitro Chondrogenesis

After monolayer expansion through two passages in the absence or presence of FGF-2, the cells were trypsinized and resuspended at 5 × 10^5^ cells/mL in chondrogenic medium (high-glucose, glutamine-sodium pyruvate-DMEM containing 5 ng of TGF-*β*1/mL, 37.5 *μ*g of ascorbic acid/mL, 10^−7^ M dexamethasone, 6.25 *μ*g of insulin/mL, 6.25 *μ*g of transferrin/mL, 6.25 ng of selenite/mL, 300 *μ*g of L-glutamine/mL, 100 U of sodium penicillin/mL, and 100 *μ*g of streptomycin sulfate/mL). Five hundred microliters of medium, containing 2.5 × 10^5^ cells, was centrifuged at 390 rfu for 5 min in 1.5 mL microcentrifuge tubes. The caps of the microcentrifuge tubes were punctured with an 18G needle after pelleting to allow gas exchange. After 3 days in the centrifuge tubes, the pellets were gently aspirated from the tubes and transferred to 6- or 24-well ultralow attachment culture plates (Corning Inc., Corning, NY). Pellets were maintained in chondrogenic medium, with changes every 48–72 hours. On days 10 and 20, a single representative pellet in each group was fixed in 4% paraformaldehyde for histologic processing. The remaining pellets were snap-frozen in liquid nitrogen and stored at −80 degrees Celsius for further analyses.

### 2.6. Pellet DNA Content

The Hoechst fluorescence assay [35] was used to measure DNA content of the pellets. Three pellets were digested in 250 *μ*L of papain digest (0.15 mg/mL; SIGMA Chemical MPC, St. Louis, MO) for 16 hours at 65°C. Serial dilutions of calf thymus DNA were used to generate a standard curve. Duplicate 10 *μ*L aliquots of each sample and standard were pipetted into black 96-well microplates. Hoechst 33258 fluorescent dye was added in each well and the optical density was measured at 485 nm wavelength (FLUOstar Optima Microplate Reader, BMG LABTECH, Durham, NC). The values were adjusted to “*μ*g of DNA per pellet.”

### 2.7. Pellet Collagen Type II Content

Three pellets from each treatment group/time point were digested in 50 *μ*L of pepsin-acetic acid (0.5 mg/mL) at 4°C overnight, with continuous mixing on a rotator. The day after, the pellets were transferred to an elastase digestion solution (1 mg/mL pancreatic elastase in 1x TBS) for 24 h. A commercial ELISA assay was used to measure collagen type II protein in each sample, following the manufacturer's recommended protocol (Chondrex Inc., Redmond, WA). Briefly, 100 *μ*L of capture antibody solution was pipetted in each well of 96-well plates and incubated at 4°C overnight. The next day, the wells were washed before adding 50 *μ*L of the sample digests and type II collagen standards. After 2 hours of incubation at room temperature, the detection antibody solution (50 *μ*L) was added to each well, followed by a second incubation. Streptavidin peroxidase solution (100 *μ*L) was then added, followed by a one-hour incubation. Lastly, 100 *μ*L of chromatin dilution buffer solution was added to each well. After 30 min of incubation, 50 *μ*L of stop solution (2N sulfuric acid) was added to each well and the optical densities were measured spectrometrically at 405 nm using a FLUOstar Optima Microplate Reader (BMG LABTECH, Durham, NC). The collagen type II values were converted to “*μ*g/pellet.”

### 2.8. Pellet Sulfated Glycosaminoglycan Content

The dimethyl methylene blue dye-binding (DMMB) assay was used to measure sulfated glycosaminoglycans (sGAG) in the pellets [36]. Three pellets from each treatment/time group were digested in 250 *μ*L of 0.15 mg/mL papain digestion buffer (SIGMA Chemical MPC, St. Louis, MO) for 16 hours at 65°C overnight. The next day, the samples were digested with DNAse at 37°C for 20 minutes. Two hundred microliters of DMMB reagent was added to 50 *μ*L of the digested samples and optical densities were measured at 530 nm (FLUOstar Optima Microplate Reader, BMG LABTECH, Durham, NC), along with serial dilutions of chondroitin sulfate standards. The “sGAG” values were expressed as “*μ*g sGAG/pellet.”

### 2.9. RNA Isolation, Reverse Transcription, and PCR Amplification

Total RNA was extracted using a commercial guanidinium thiocyanate-phenol reagent (Trizol, Invitrogen Corp., Life Technologies, Grand Island, NY), following the manufacturer's instructions. The isolates were purified over silica columns (RNeasy, Qiagen Inc., Hilden, Germany). One microgram of total RNA from each sample was reverse-transcribed (Superscript II, Invitrogen Corp., Life Technologies, Grand Island, NY), using standard protocols and oligo-dT primers.

Gene-specific primers for collagen type II, aggrecan, alkaline phosphatase (ALP), collagen type X, Sox9, Mef2C, and Runx2 (Table 1) were designed from available published sequences in Genbank and using ClustalW multiple sequence alignment (available at http://www.ebi.ac.uk/) and Primer 3 software (http://bioinfo.ut.ee/primer3-0.4.0/). Primer specificity was confirmed by melt curve specificities and by cloning and sequencing the amplicons during optimization experiments. PCR amplifications were catalyzed by Taq DNA polymerase (BioRad iCycler, BioRad Laboratories, Hercules, CA) in the presence of Sybr green. Relative gene expression was quantified using the 2^−∆∆CT^ method [37], corrected for amplification efficiencies, and normalized to expression of the reference gene, elongation factor-1*α* (EF1*α*).

### 2.10. Histologic Examination

One representative pellet from each treatment/time group was fixed in 4% paraformaldehyde for 24 hours. The pellets were then immobilized in cassettes using HistoGel (Richard-Allan Scientific, Radnor, PA), transferred to PBS solution, and stored at 4°C. The pellets were dehydrated in alcohol, embedded in paraffin, sectioned at 8 *μ*m, and stained with Toluidine Blue. Histological images were acquired using 20x and 40x objectives, utilizing the Nanozoomer 2.0 HT Digital Pathology System machine (Hamamatsu Photonics K.K., Hamamatsu, Japan).

### 2.11. Statistical Analyses

The normality of distribution of the quantitative data (monolayer proliferation, pellet DNA content, pellet sGAG content, pellet collagen type II content, and relative mRNA expression) was confirmed using the Shapiro-Wilk test, the Bell histogram, and Normal Q-Q plot (IBM SPSS Statistics). The data were expressed as “mean ± standard deviation.” Paired Student's *t*-tests were used to assess the effects of FGF-2 on population doubling and population doubling times. Two-way repeated measures ANOVA was used to assess the effect of FGF-2 across time on cell proliferation, pellet DNA content, pellet sGAG content, pellet collagen type II content, and relative mRNA expression. A *p* value of ≤ 0.05 was considered significant.

## 3. Results and Discussion

### 3.1. Results

#### 3.1.1. Cell Expansion

**Figure 1:**
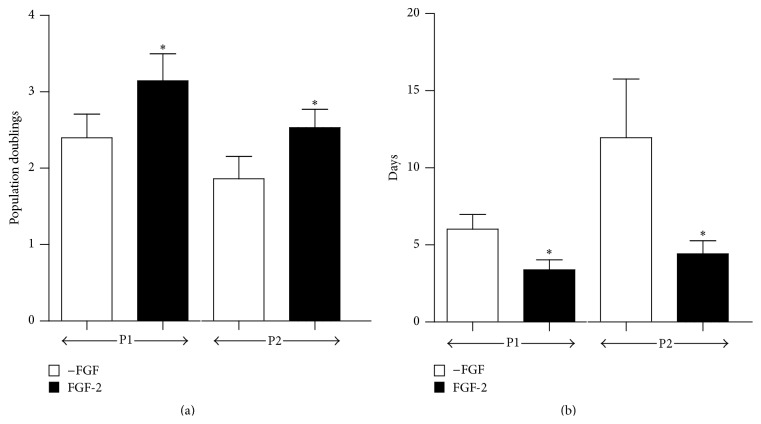
Effect of FGF-2 supplementation on SF-CP proliferation. (a) Population doublings and (b) population doubling (PD) times, during the first (P1) and second (P2) passages, in the absence (white bars) or presence (black bars) of FGF-2 (mean ± SD; *n* = 15). In both figures, asterisks indicate significant differences between the control and FGF-treated cultures at each passage.

There were, on average, 59.2 CFUs/mL of synovial fluid (range 25.2–178.7 CFUs/mL; *n* = 11) in primary cultures of aspirates. The time between initial seeding of the aspirates and near-confluence of the primary cultures was 17.1 ± 5.2 days. Supplementing media with FGF-2 significantly increased population doubling during both the first (2.59 ± 1.29 in control cultures versus 3.34 ± 1.43 in FGF-2 cultures; *p* = 0.013, *n* = 15) and second (1.86 ± 1.13 in control cultures versus 2.53 ± 0.93 in FGF-2 cultures; *p* = 0.063, *n* = 15; Figure 1(a)) passages. Accepting the variation in responses, this represents an approximate 1.6-fold increase in cell numbers in response to FGF-2 during both passages. FGF-2 also significantly reduced the population doubling times (Figure 1(b)). During the first passage, control cultures required 5.6 ± 3.60 days for each population doubling, while cultures treated with FGF-2 required approximately half this time (2.88 ± 1.93 days; *p* = 0.02, *n* = 15). During the second passage, the mean population doubling time in control cultures increased to 10.25 + 9.25 days. Again, FGF-2 administration reduced the doubling time by approximately 50% (4.48 ± 3.42 days) in the second passage cultures.

#### 3.1.2. Immunophenotypic Analysis

**Figure 2:**
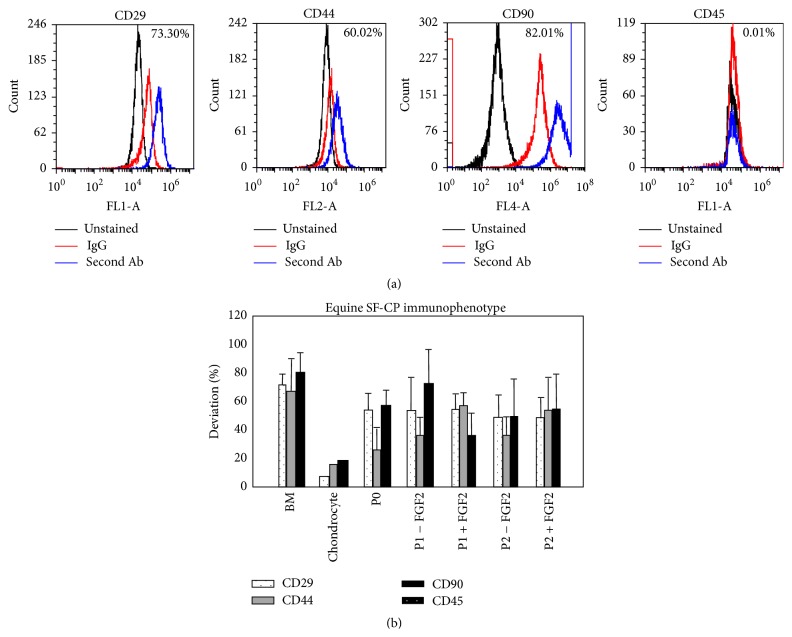
SF-CP immunophenotype characterization. (a) Representative immune-phenotypic profile of passage 1 SF-CPs (horse 2), supplemented with FGF-2. (b) Immunophenotypic characterization (mean ± SD) of SF-CPs at passages 0, 1, and 2, along bone marrow (BM) MSCs and chondrocyte control populations (*n* = 8).

SF-CPs were immunopositive for the three surface cell markers CD29, CD44, and CD90 that characterize equine MSCs [33, 34] and negative for the hematopoietic marker, CD45. FGF-2 administration did not affect the immunophenotype of SF-CPs during monolayer expansion (Figure 2). Further, the immunophenotype of SF-CPs did not change significantly across passages in either group.

#### 3.1.3. Pellet DNA Content

**Figure 3:**
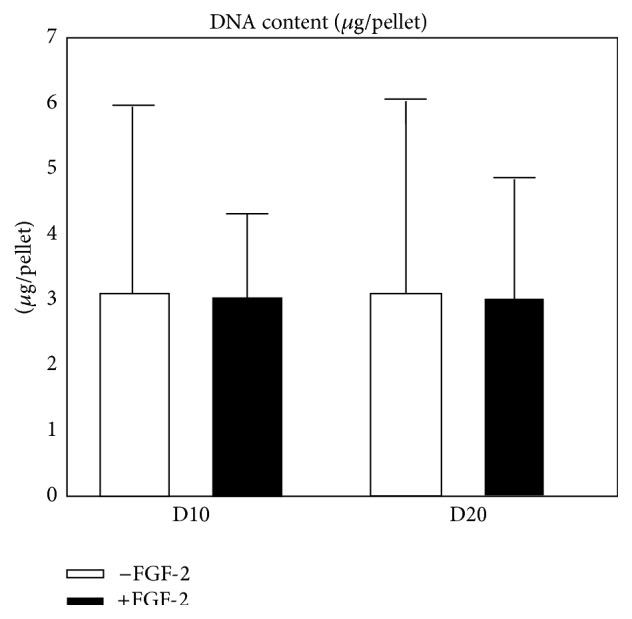
DNA content (*μ*g/pellet) on days 10 and 20 of chondrogenic culture, with (white) or without (black) FGF-2 supplementation (*n* = 6).

Monolayer expansion of SF-CPs in the presence of FGF-2 had no significant “carry over” effect on the DNA content of chondrogenic pellets at either time point (Figure 3; *p* = 0.913; *n* = 6). In both the control and FGF-2 pellets, DNA contents were stable at approximately 3 *μ*g/pellet throughout the time course of the experiments.

#### 3.1.4. Chondrogenic Gene Expression

**Figure 4:**
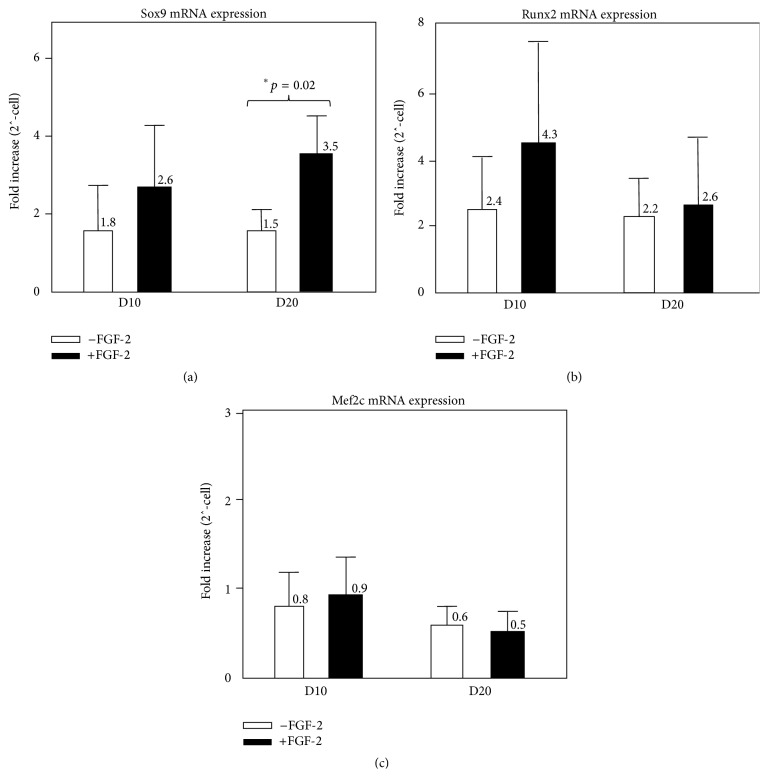
Sox9 (a), Runx2 (b), and Mef2c (c) mRNA expression (fold increase) on days 10 and 20 of chondrogenic culture with (white) or without (black) FGF-2 supplementation (*n* = 3; 3; 3).

**Figure 5:**
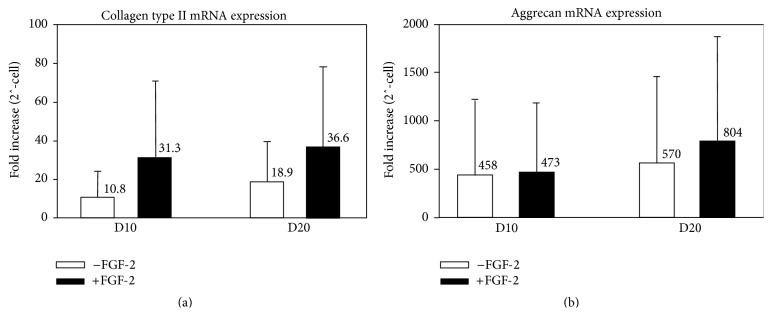
Collagen type II (a) and aggrecan (b) mRNA expression (fold increase) on days 10 and 20 of chondrogenic culture with (white) or without (black) FGF-2 supplementation (*n* = 5; 5).

FGF-2 significantly increased Sox9 mRNA levels expression on day 20 (1.5 ± 0.6-fold increase in control pellets versus 3.5 ± 1.05-fold increase in FGF-2 pellets, *p* = 0.02; Figure 4(a)) but had no effect on expression of the transcription factors Runx2 and Mef2c (Figures 4(b) and 4(c)), both required for hypertrophic differentiation [38–41]. FGF-2 increased collagen type II mRNA levels 2-3-fold on day 10 (10.8 ± 13-fold increase in control pellets versus 31.3 ± 39-fold increase in FGF-2 pellets) and day 20 (18.9 ± 20.7-fold increase in control pellets versus 36.6 ± 40.7-fold increase in FGF-2 pellets); however these differences were not statistically significant, due to high interdonor variability (Figure 5(a)). Steady state aggrecan mRNA levels increased approximately 500-fold by day 10 in both the control (458 ± 765-fold increase) and FGF-2 (473 ± 726-fold increase) groups, in comparison to undifferentiated SF-CPs, and this upregulation was sustained on day 20. FGF-2 had no significant effect on aggrecan expression (Figure 5(b)).

**Figure 6:**
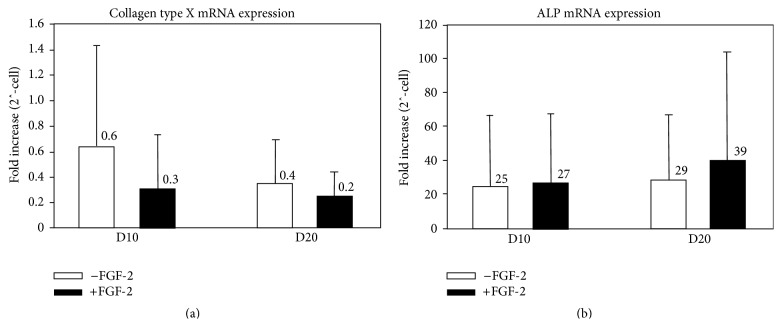
Collagen type X (a) and ALP (b) mRNA expression (fold increase) on days 10 and 20 of chondrogenic culture with (white) or without (black) FGF-2 supplementation (*n* = 3; 5).

Collagen type X and ALP transcript levels were extremely low in all the pellet experiments (threshold cycles were routinely 8–10 higher than in cell populations capable of hypertrophic differentiation; Figures 6(a) and 6(b)). In control pellets, collagen type X transcript levels fell throughout the 20-day culture period (0.63 ± 0.81-fold on day 10 and 0.37 ± 0.36-fold on day 20), while ALP mRNA levels increased only slightly (25.3 ± 42.67 on day 10 and 29.3 ± 38.33 on day 20). FGF-2 did not significantly alter expression of collagen type X (0.33 ± 0.43-fold on day 10 and 0.25 ± 0.20-fold on day 20) or ALP (28.0 ± 42.67-fold on day 10 and 39.33 ± 66.67-fold on day 20), consistent with the Mef2c and Runx2 results.

#### 3.1.5. Pellet Matrix Content

**Figure 7:**
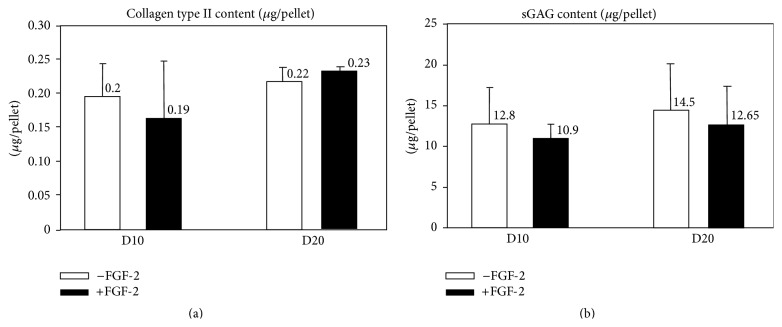
Collagen type II (a) and sGAG (b) proteins content (*μ*g/pellet) on days 10 and 20 of chondrogenic culture with (white) or without (black) FGF-2 supplementation (*n* = 5).

FGF-2 administration did not affect collagen type II protein (on day 10: 0.20 ± 0.04 *μ*g/pellet in control pellets versus 0.19 ± 0.06 *μ*g/pellet in FGF-2 pellets; on day 20: 0.22 ± 0.02 *μ*g/pellet in control pellets versus 0.23 ± 0.01 *μ*g/pellet in FGF-2 pellets) or sGAG (on day 10: 12.8 ± 4.5 *μ*g/pellet in control pellets versus 10.9 ± 1.8 *μ*g/pellet in FGF-2 pellets; on day 20: 14.5 ± 5.7 *μ*g/pellet in control pellets versus 12.65 ± 4.7 in FGF-2 pellets) contents in the pellets at either time point (Figures 7(a) and 7(b)), consistent with the qPCR results.

#### 3.1.6. Pellet Histology

**Figure 8:**
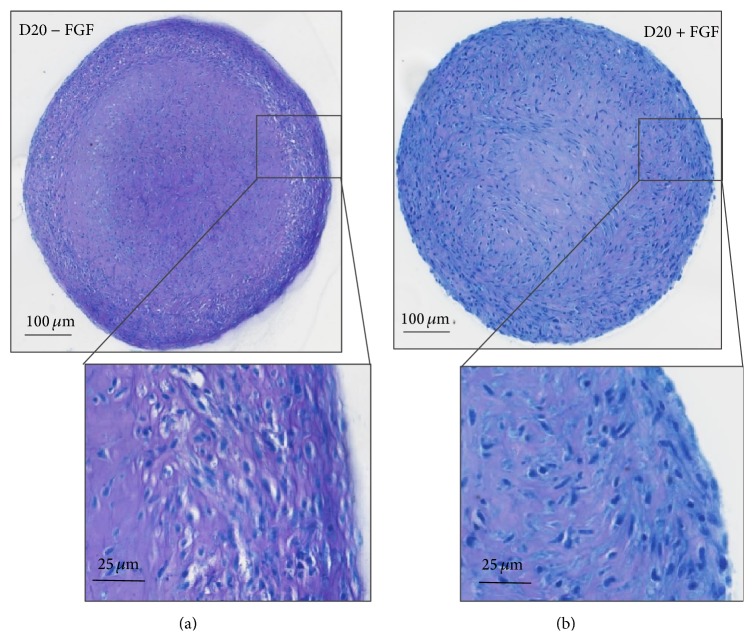
Histological sections of chondrogenic pellets on day 20. Pellets from cells expanded in control medium (a) or medium supplemented with FGF-2 (b) were stained with Toluidine Blue.

Toluidine Blue staining intensity, reflecting sGAG content, was not affected by FGF-2 administration during monolayer expansion. Overall pellet size was also unaltered. It was notable, however, that FGF-2 supplementation prevented the development of a flattened “fibroblastic” cell layer that occupied the peripheral 100 *μ*m of control pellets (Figure 8). There were no indications of central hypertrophic differentiation in either group, consistent with the qPCR data.

### 3.2. Discussion

Consistent with our previous studies [26] and the reports from several other groups [18–25], equine SF-CPs were capable of considerable in vitro proliferation (Figure 1) and subsequent chondrogenic differentiation (Figures 7 and 8). There was considerable variation in the number of CFUs per mL of synovial fluid and the time required for establishing primary SF-CP cultures. These results are consistent with other reports [19, 20, 22] and are likely influenced by the initial uneven distribution of clonal cell groups across the plate surfaces and consequent variation in local cell densities. In light of this, the “80% confluence” designation for primary culture passages should be considered a nominal value.

Monolayer expansion in medium supplemented with FGF-2 significantly increased population doubling and halved the population doubling times during both passages. In this respect, the hypothesis addressing FGF-2's effect on SF-CP proliferation is accepted. This potent mitogenic effect has also been reported in several previous studies in human bone marrow-derived stem cells [30, 42–45] with 2-3-fold increases in proliferation rates being reported. FGF-2 exerts its mitogenic effects via the MAPK signaling pathway [30, 42, 46], accelerating transit through the G1 phase of the cell cycle [47]. The proliferative activity of the control cultures during passage 2 was noticeably less than during passage 1, reflected by reduced population doublings and a twofold increase in the PD time (Figure 1). Although all control cultures did reach confluence during P2, these outcomes suggest that control cultures were approaching senescence. Proliferative failure was reported by Kurose et al., 2010, in six of 25 synovial fluid samples from human knee OA patients [20]. FGF-2 supplementation slows the development of senescence in proliferating bone marrow-derived stem cell populations [48–50], and it is highly likely that FGF-2 influences progenitor cells from synovial fluid similarly. Collectively, the increased population doublings during passage and reduced population doubling times stimulated by FGF-2 mitigate a major obstacle to using of SF-CPs for potential clinical applications, such as intrinsic cartilage repair, tissue engineered cartilage, and the immune-modulation of inflammatory arthritis [20, 28, 29].

Accepting species differences in progenitor cell immunoprofiles, the cell surface marker profiles from equine SF-CPs were consistent with results from other studies [20, 27–29] and were characteristic of equine MSCs (CD29+, CD44+, CD90+, and CD45−; [33, 34]). The impact of FGF-2 on SF-CP proliferation did not negatively influence the immunophenotype of the expanded cell populations. Of particular interest, the relative expression of these stem cell markers did not change significantly during multiple passages, suggesting that there was no “enrichment” process occurring through selective stem cell proliferation. Rather, the immunophenotypic consistency across passages suggests that SF-CPs were the predominant cell type engaged in population expansion in the primary cultures and subsequent passages.

FGF-2 administration during monolayer expansion did not negatively impact subsequent SF-CP chondrogenesis, despite the reported detrimental effects of prolonged expansion on MSC chondrogenic capacity [42] and the more general “dedifferentiating” effects prolonged monolayer expansion exerts on the chondrocytic phenotype [5, 6]. FGF-2 increased steady state mRNA levels of the chondrogenic transcription factor, Sox9, on day 20, and both collagen type II and aggrecan transcripts were also increased by FGF-2, although not to a statistically significant degree. Despite these effects, FGF administration did not alter collagen type II protein or sGAG deposition within the pellet matrices. In light of these outcomes, the hypothesis addressing FGF-2's effect on SF-CP chondrogenesis is rejected. This “disconnection” between transcriptional and translational productivity is a common observation in stem cell/tissue engineering biology and indicates that the biosynthetic capacities of newly in vitro differentiated MSCs do not match those of fully differentiated cell populations [42, 51]. This limitation will need to be resolved for stem cell applications to be successful in tissue engineering applications.

Although there was no quantitative effect on cartilage matrix production, FGF-2 clearly improved the histological characteristics and cytomorphology of the chondrogenic pellets, preventing the development of a zone of flattened cells around the pellet surface, approximately 100 *μ*m deep (Figure 8). This peripheral zone of flattened “dedifferentiated” or “perichondral” cells is a consistent feature of MSC and chondrocytic pellet culture models [6, 29, 30, 32, 42, 52] and is considerably more substantial than the flattened superficial zone of mature articular cartilage [53]. The absence of this feature in pellets from FGF-treated SF-CPs suggests that expansion in the presence of FGF-2 generates a phenotypic homogeneity in the expanded population that is not present in populations expanded in FBS alone.

Chondrogenic equine SF-CPs do not express hypertrophic chondrocytic markers (collagen type X or ALP) under control culture conditions, and FGF-supplementation did not affect this. The threshold cycles for these genes in the SF-CP samples were routinely between 8 and 10 cycles (2-3 logs) higher than thresholds in cell populations (such as growth plate chondrocytes and bone marrow-derived MSCs) undergoing robust hypertrophy (data not shown). This “nonhypertrophic” phenotype lends credence to the use of SF-CPs for articular cartilage repair applications, since the phenotypic match is far closer than with other MSC sources.

The source(s) of SF-CPs has not yet been definitively determined. Chondroprogenitors are present in the subchondral bone marrow compartment and, under appropriate pathological conditions [54], migrate into fibrillated cartilage and the joint space. However, there were no overt arthritic changes in the joints of the horses used in this study, and increased numbers of SF-CPs were found in the synovial fluids of early arthritic disease cases in people [18], prior to the development of overt cartilage fibrillation or penetration into the subchondral bone space. Comparative gene expression profiling by Morito et al., 2008 [21], and by Sekiya et al., 2012 [22], strongly suggested that SF-CPs are derived from the synovium, rather than the bone marrow compartment, and this possibility is also supported by the findings of Jones et al., 2008 [18], in that the number of synovial fluid CFUs correlated with the prevalence of microscopic synovial tissue fragments in the fluid aspirates, and of Zhang et al., 2004, who demonstrated that synovial fluid contains chemotactic factors that recruit stem cells from osteoarthritic synovium [55]. In severely pathological joints, it is possible that progenitors in synovial fluid originate from several intra- and periarticular tissue sources, the synovium [21, 22, 57], subchondral bone space [54], and the articular cartilage itself [55, 56]. Future research should focus on identifying the sources of SF-CPs and developing strategies to utilize these cells to support articular cartilage homeostasis and repair.

## 4. Conclusions

FGF-2 significantly increased SF-CPs in vitro expansion, significantly increasing population doublings and reducing population doubling times. FGF-2 did not affect the immunophenotype of SF-CPs during expansion or compromise subsequent SF-CP chondrogenesis. FGF-2 did prevent the development of a flattened “fibroblastic” cell layer around the periphery of the pellets indicating a phenotypic homogeneity in the expanded cell populations. FGF-2 supplementation of SF-CP monolayer cultures significantly accelerates population expansion prior to subsequent clinical applications for articular cartilage repair.

## Figures and Tables

**Table 1:** Primers used in the qPCR reactions.

Gene (amplicon size)		Primers	Annealing temperature
EF1-alpha (328 bp)	S	5′ CCCGGACACAGAGACTTCAT	62.1°C
	A	5′ AGCATGTTGTCACCATTCCA	

Col II (223 bp)	S	5′ AGCAGGAATTTGGTGTGGAC	62.1°C
	A	5′ TCTGCCCAGTTCAGGTCTCT	

Col X (244 bp)	S	5′ TGCCAACCAGGGTGTAACAG	62.1°C
	A	5′ ACATTACTGGGGTGCCGTTC	

ALP (260 bp)	S	5′ CCACGTCTTCACATTTGGTG	54.2°C
	A	5′ AGACTGCGCCTGGTAGTT	

Aggrecan (202 bp)	S	5′ GACGCCGAGAGCAGGTGT	62.1°C
	A	5′ AAGAAGTTGTCGGGCTGGTT	

Sox9 (304 bp)	S	5′ GAACGCACATCAAGACGGAG	56.2°C
	A	5′ CTGGTGGTCTGTGTAGTCGT	

Mef2c (55 bp)	S	5′ CCCAACTTTGAGTGCCAGT	55.3°C
	A	5′ ATGTGAGGTCTCCACCCATC	

Runx2 (115 bp)	S	5′ CAGACCAGCAGCACTCCATA	56.8°C
	A	5′ GAGCGTCAACACCATTC	
